# Online educational interventions in pediatric intensive care medicine

**DOI:** 10.3389/fped.2023.1127754

**Published:** 2023-03-09

**Authors:** Dennis Daniel, Traci A. Wolbrink

**Affiliations:** Department of Anesthesiology, Division of Critical Care Medicine, Critical Care and Pain Medicine, Department of Anaesthesia, Harvard Medical School, Boston Children's Hospital, Boston, MA, United States

**Keywords:** online education, video-based learning, medical education, interactive learning, distance education

## Abstract

**Background:**

Online education has experienced explosive growth, particularly in the wake of the COVID-19 pandemic. We explored the current state of the evidence base for online education targeted towards healthcare professionals working in pediatric intensive care units (PICUs), to report how we are using online education in our field.

**Materials and Methods:**

We performed a literature review by systematically generating a list of publications indexed in PubMed describing online educational interventions in the PICU, using Medical Subject Header (MeSH)-based search terms and the following inclusion criteria: studies published after 2005 that describe online educational interventions aimed at healthcare professional working in the PICU. We reviewed the full text of all included articles, and summarized the study aims, design, and results.

**Results:**

Our initial search yielded 1,071 unique articles. After screening abstracts and titles, then full texts, eight articles were included in the review. Many online learning modalities are represented, including websites, self-study modules, videos, videoconferencing, online self-assessment with feedback, virtual patient cases, screen-based simulation, and podcasts. Three studies focused on residents, two studies on nurses, two studies on a multidisciplinary team, and one study on transport nurses and paramedics. Most studies utilized participant surveys to assess satisfaction, and half included pre- and post-intervention multiple-choice question tests. Only one study included a patient-related outcome measure.

**Conclusions:**

Despite growth in online medical educational intervention research, there are relatively few published studies in pediatric critical care, and only one study evaluated the impact of online learning on patient outcomes. There remain significant opportunities for PICU educators to assess the impact of online educational interventions, especially related to clinician behaviors and patient outcomes.

## 1. Introduction

Both within and outside healthcare, online communication and education have become standard practice. Driven by explosive growth of the Internet, the overall volume of medical knowledge, and most recently by the 2019 SARS-CoV-2 (COVID-19) global pandemic (1–3), how we learn and teach medicine has evolved. Although traditional, in-person learning experiences remain prevalent in the ICU (4), the widespread availability of mobile computing, high-speed Internet connectivity, and a wide range of content distribution platforms creates opportunities that did not exist 30 years ago. In the pediatric intensive care unit (PICU), learners must maintain a broad, updated fund of knowledge, and be prepared to apply it in time-sensitive, pressured situations as part of a multidisciplinary care team. Online approaches to education and information sharing can offer powerful approaches to meeting a variety of learning needs in this acute, complex patient care environment.

Online education activities include any activities that are delivered *via* the Internet and that inspire medical learning. The number and variety of online experiences and content delivery platforms is always shifting. Catalogues of recommended online medical education platforms and resources are readily available in the literature. One such list featuring resources specific to intensive care was published in 2019 (5). Previous reviews have ratified the ability of online medical education approaches to have a beneficial impact on learning outcomes relative to no education, and a comparable impact to more classical approaches (6). Although there are many described examples of technology-enhanced medical educational interventions, relatively few of these describe interventions specifically targeting the PICU context.

In this article, we aim to explore the current state of the evidence base for online education targeted towards healthcare professionals working in the PICU. We present the results of a literature review with a goal to provide pediatric intensive care clinicians with an updated awareness of the state of educational evidence for online education in our field, to promote understanding of how and when online approaches to education may be beneficial in the clinical learning environment.

## 2. Materials and methods

We performed a literature review by systematically generating a list of publications indexed in PubMed that describe online educational interventions in the PICU. Searches were conducted using Medical Subject Header (MeSH) terms submitted to the PubMed search engine. We combined the MeSH terms “Intensive Care Units, Pediatric” and “Critical Care” with the following MeSH terms and Boolean operators: AND Education, Distance; AND Computer-Assisted Instruction; AND Computer Simulation; AND Simulation Training; AND Instructional film and video; AND Teaching; AND Gamification; AND Internet-Based Intervention. The full list of MeSH terms used and number of search results returned is provided in Supplementary S1.

All titles and abstracts were manually screened by both authors independently, using the following inclusion criteria: published on or after January 1, 2005; abstract available in English; manuscript includes description of a medical educational intervention with online or computer-enabled components; study includes healthcare professionals as learners; intervention includes PICU or pediatric cardiac ICU setting; includes at least one outcome measure. Exclusion criteria were: educational intervention involves only manikin-based simulation; included patients or caregivers as learners; intended for use exclusively in the neonatal or adult ICU patient population or setting; or editorial format. After initial screening, the two authors developed complete consensus regarding the included abstracts. Finally, full texts were then screened, and the two authors developed complete consensus regarding the included studies.

## 3. Results

**Figure 1:**
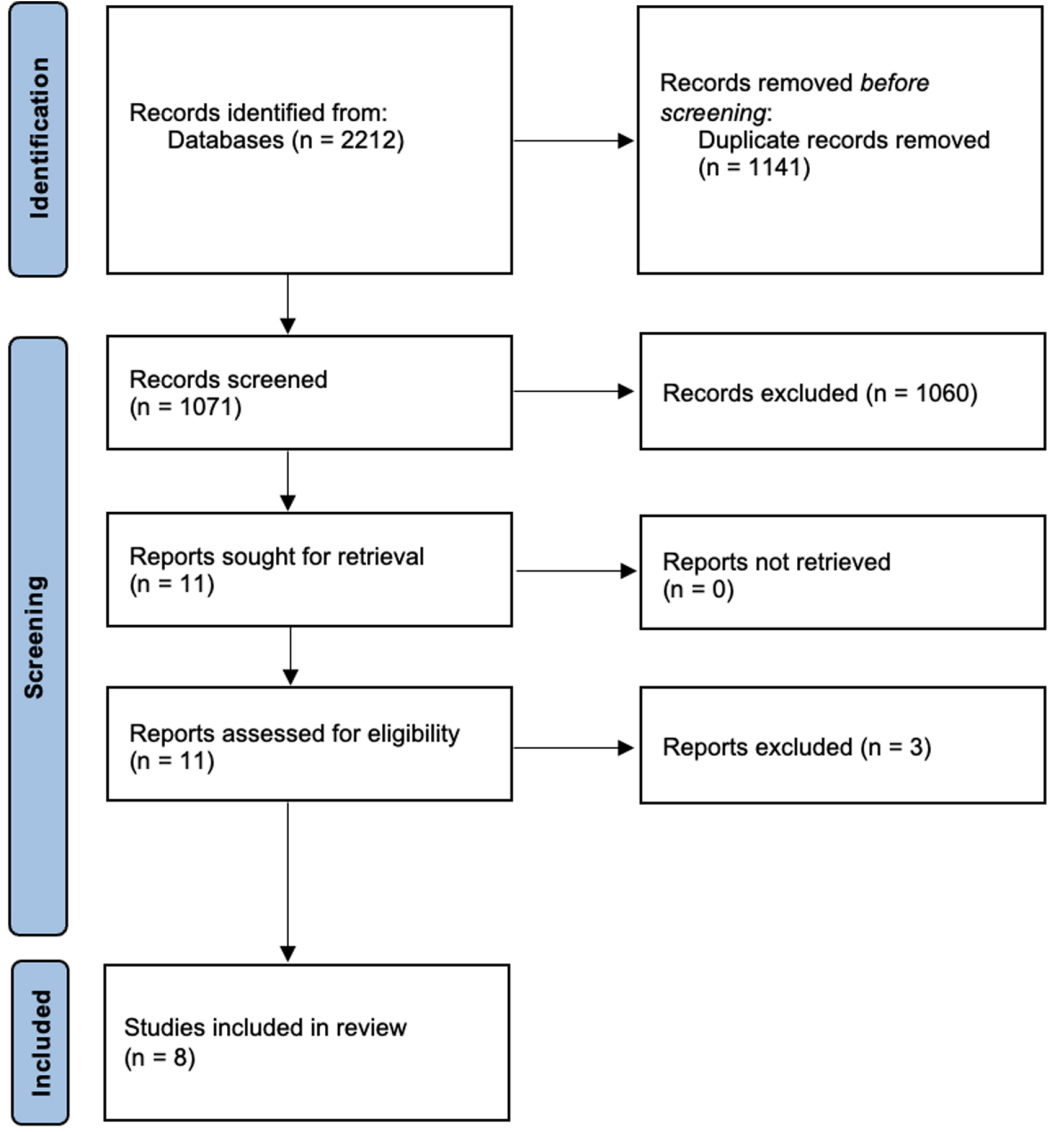
The PRISMA diagram reporting the results of our search and screening process.

We identified 1,071 articles in our initial search, and eight articles met inclusion criteria and were subsequently evaluated for this study. The PRISMA diagram reporting the results of our search and screening process is provided as Figure 1. The eight manuscripts selected for inclusion are listed in Table 1 and include the author, title, description of the intervention, outcomes measures, and key findings. The majority of the initial search results were excluded from further review on the basis of not including any online educational intervention, or exclusively involving neonatal or adult ICU contexts. Of the eleven manuscripts that underwent full review, one was excluded for not involving the PICU context (7), and two were excluded for lacking an educational intervention (5, 8).

**Table 1:** The author, title, description of the intervention, outcomes measures, and key findings are summarized for the eight manuscripts selected for inclusion.

Author	Title	Description of intervention	Outcomes Measured	Findings
Bursch et al.	Feasibility of Online Mental Wellness Self-assessment and Feedback for Pediatric and Neonatal Critical Care Nurses	Pediatric and neonatal intensive care unit nurses completed anonymous online self-assessments of burnout, resilience, trauma, depression, anxiety, and workplace stress, and received immediate feedback and education.	Participant survey of satisfaction; ability of survey variables to estimate prevalence of psychiatric symptoms and predict burnout.	Most participants found the online assessment and feedback to be helpful. Longer nursing experience and older age were associated with lower levels of reported anxiety, and choosing nursing as a second career was associated with higher resilience.
Daniel et al.	Pediatric Resident Engagement With an Online Critical Care Curriculum During the Intensive Care Rotation	This study aimed to characterize the patterns of engagement with an online video-based PICU learning curriculum among pediatric residents at two clinical sites during their one-month PICU rotation.	Overall curriculum completion; time of engagement with the curriculum as reflected by time of completing pre- and post-lesson assessments, in relation to clinical duty, clinical schedule type (rotating 24-hour call vs. day/night shifts), time of day, and day of rotation.	Curriculum completion was not associated with clinical schedule type. Residents mainly completed the curriculum between 6PM and 6AM, whether or not they were on clinical duty. Roughly two-thirds of overall curriculum engagement occurred while residents were not scheduled for clinical duty.
Good et al.	Point-of-Care Ultrasound Education for Pediatric Residents in the Pediatric Intensive Care Unit	The authors created a bundled educational intervention teaching basics of point-of-care ultrasound (POCUS) to pediatric residents completing their one-month PICU rotation, consisting of three didactic modules to be completed *via* self-study, accompanied by pre- and post-course assessments, and guidelines for conducting small group hands-on POCUS sessions and bedside POCUS educational rounds.	Participant survey of satisfaction and self-efficacy; demonstrated knowledge before and after the curriculum; comparison of post-course test scores with test scores from a historical cohort of residents who completed the rotation but not the POCUS curriculum.	Participants completing the curriculum reported increased knowledge of POCUS and comfort performing POCUS, and were satisfied with the small group and bedside rounds sessions. Post-curriculum test scores were higher than the pre-curriculum scores for five out of six included residents. Residents completing the curriculum had higher test scores compared to the historical cohort.
LaFond et al.	Development and validation of a virtual human vignette to compare nurses’ assessment and intervention choices for pain in critically ill children	This study describes the development and validation of four virtual cases to be used for nursing assessment of pain in critically ill children.	Responses to surveys rating the virtual patient's level of pain and semi-structured interviews about relevance to practice by PICU nurses.	PICU nurses reported vignette consistency with clinical practice. Facial expression recognition was high. Nurses gave lower pain scores to smiling patients as compared to grimacing patients.
Leviter et al.	Point-of-Care Ultrasound Curriculum for Endotracheal Tube Confirmation for Pediatric Critical Care Transport Team Through Remote Learning and Teleguidance	The authors developed a multimodal curriculum including videos, podcasts, readings, and videoconferencing for remote coaching and case-based discussion to teach pediatric critical care transport team nurses and paramedics the use of point-of-care ultrasound (POCUS) for confirmation of appropriate endotracheal tube (ETT) placement.	Participant survey of satisfaction and attitudes relating to POCUS; demonstrated knowledge on pre- and post-curriculum assessment tests; retention of knowledge and hands-on POCUS skills at 2 weeks and 3 months post-intervention; number of independent POCUS studies performed following the intervention.	Participants demonstrated both knowledge and skill acquisition relating to the use of POCUS for ETT placement confirmation, and reported comfort with POCUS and willingness to use it in the future. Participants were satisfied with the online curriculum. Test scores at 2 weeks and 3 months post-curriculum were the same or higher compared to both pre-curriculum and immediately post-curriculum. Independent POCUS use by participants in the course of clinical work remained low, increasing from 0% to 7.4% of all cases during the study period.
Polito et al.	Children's Cardiology Up-to-Date Online Resources for Education (CUORE) project: remote education for training in pediatric critical care medicine	Clinicians working in two pediatric cardiac ICU (USA and Chile) used videoconferencing to deploy five conferences related to central venous cather-associated bloodstream infections (CLABSI).	CLABSI rates in the Chilean pediatric cardiac ICU; survey to assess satisfaction with the intervention.	There was no difference in CLASBI rates following the intervention. Most participants felt the topics were relevant to practice, aimed at an appropriate level, and would prefer to maintain the intervention format.
Wolbrink et al.	Online Learning and Residents’ Acquisition of Mechanical Ventilation Knowledge: Sequencing Matters	In this multi-center trial, residents rotating through the PICU for the first time were allocated to either complete a screen-based simulator before or after their one-month PICU rotation. Mechanical ventilation knowledge was assessed before and after each intervention.	Demonstrated knowledge on multiple choice question tests before and after each intervention.	Residents who used an online screen-based simulator, gained an equivalent amount of knowledge as residents that spent one month working in a PICU; residents retained more knowledge from using the simulator as compared to one month in the PICU; and residents that used the simulator before their PICU rotation gained twice as much knowledge as residents that used the simulator after their PICU rotation.
Wolbrink et al.	The development of an internet-based knowledge exchange platform for pediatric critical care clinicians worldwide	The authors present results of an educational needs assessment survey of physicians and nurses who care for critically ill children and describe the development of an Internet-based pediatric critical care medicine educational application including videos, protocols, multiple-choice quizzes, and an interactive mechanical ventilation simulator.	Educational needs as expressed on the needs assessment survey.	Survey respondents represented 49 countries. Half of respondents reported using the Internet at least once per week to obtain professional education. The most frequently requested topics were respiratory care (mechanical ventilation), sepsis, neurology, cardiology, extracorporeal membrane oxygenation, and ethics. An Internet-based educational application containing information addressing the expressed needs was successfully developed and launched.

There are a variety of online learning modalities represented in the articles including: websites, self-study modules, videos, videoconferencing, online self-assessment with feedback, virtual patient cases, screen-based simulation, and podcasts. Three of the included studies (9–11) describe interventions that apply more than one of the presentation modalities listed. No virtual or augmented reality interventions for healthcare professional education in pediatric intensive care were identified through this review. Three studies (9, 12, 13) included interventions aimed at residents rotating in the PICU, two studies (14, 15) included interventions aimed at nurses, two studies (11, 16) included interventions aimed at a multidisciplinary healthcare professionals, and one study (10) included an intervention aimed at transport nurses and paramedics. In terms of outcome measures analysed, the majority (6 of 8) of studies utilized participant surveys to assess satisfaction or change in attitudes (9–11, 14–16), and nearly half (4 of 8) included pre- and post-intervention multiple-choice question tests (9, 10, 12, 13). Only one study included a patient-related outcome measure (16).

The broad range of educational content that *websites* can present is familiar to most casual users of the Internet, ranging from static text to highly complex, detailed, and fully interactive experiences. Many websites also provide platforms for presentation, curation and distribution of materials produced by others. From the standpoint of medical education, there are more options than ever before for medical learners to individualize their educational experiences throughout their training and subsequent careers. One article describes the development of OPENPediatrics, a free website for pediatric healthcare professionals (11).

Educational *videos* have become a mainstay of education (17), including medical education (18, 19). A diverse range of tools now exists to facilitate educational video creation. Beyond the video itself, the learning experience may be improved through the inclusion of active learning through test-enhanced strategies (20, 21). Two articles specifically discussed the use of videos. One article described a video-based learning curriculum for pediatric residents rotating in the PICU (12). In this prospective study of engagement and PICU knowledge gain with video-based curricula across two institutions, most rotating residents completed the curriculum during night hours, even outside of clinical duty. This finding highlights the circumspection required when implementing blended learning curricula for trainees, particularly in the PICU environment, due to the risk of increasing total workload in a manner that risks interference with appropriate rest and recovery. One article (10) used video as part of a blended learning strategy for point-of-care ultrasound training (described in further detail below).

The use of *videoconferencing* and virtual classroom platforms has increased in recent years across settings both within and outside medical education (22, 23), and has been particularly accelerated recently by requirements for physical distancing, quarantine and isolation in association with the COVID-19 global pandemic (24–26). One included article used videoconferencing to host five conferences related to central venous catheter-associated bloodstream infections (CLABSI) between clinicians working in two pediatric cardiac ICUs in the USA and Chile (16). Although there was no difference in CLASBI rates following the intervention, most participants felt the topics were relevant to practice, aimed at an appropriate level, and would prefer to maintain the intervention format.

There is increasing interest in delivering medical learning experiences that provide a more interactive and individualized experience than is possible with the presentation of static text or pre-recorded materials. The potential level of functionality can extend from simply clicking to navigate or reveal information all the way through full-fledged educational video games. Virtual patient cases, screen-based simulations, and virtual and augmented reality represent examples of these types of learning activities. Barriers to development of ambitious interactive learning experiences include the financial costs of development and the specialized knowledge of pedagogy, graphic and user experience design, and software development required (27).

Screen*-based simulations* are self-contained learning tools that represent or recapitulate more complicated clinical settings or tools, with the intention that actions taken in the simulated environment, and their results, can be translated to clinical activity and decision making in the real world. Many screen-based simulations are designed as serious games, or games designed for purposes other than entertainment, such as education. Serious games have been shown to be at least as effective as traditional teaching or other digital education modalities (28). One article describes the use of screen-based simulation to teach principles of pediatric mechanical ventilation (13). This article describes results of a multi-center trial evaluating the educational impact of an online, screen-based mechanical ventilation simulator among residents rotating through the PICU. Participants were ramdomized to complete the screen-based simulator either before or after their one-month PICU rotation. All participants completed both the PICU rotation and mechanical ventilation simulator, with a two-month interval in between. Mechanical ventilation knowledge was assessed using pre- and post-intervention multiple-choice question tests before and after the rotation and before and after use of the ventilator. Residents who used the screen-based simulator gained an equivalent amount of knowledge to residents that spent one month working in a PICU. Residents also retained more knowledge at two months after using the simulator as compared to spending one month in the PICU, and residents that used the simulator before their PICU rotation demonstrated an increase in their test scores that was twice as large as residents that used the simulator after their PICU rotation.

Grounded in experiential learning theory, *virtual patients* are online interactive simulations of real-life patient scenarios for the purposing of practicing clinical reasoning skills. Virtual patients have been shown to be at least as effective in knowledge and skills acquisition as compared to traditional teaching (29). One article described the development and validation of four virtual cases to be used for nursing assessment of pain in critically ill children (15). In this study, patient vignettes of four patients with different medical problems and facial expressions were administered to PICU nurses. PICU nurses reported that the vignettes were consistent with experiences in clinical practice. Facial expression recognition was high, and nurses gave lower pain scores to smiling patients as compared to grimacing patients.

*Virtual reality (VR) and augmented reality* (AR) experiences use special interfaces to provide a higher level of immersion and to enable new types of interaction, most prominently including 3-dimensional spatial and kinesthetic activities. VR/AR experiences are becoming more feasible to develop and deploy outside of research contexts (30). We did not identify any articles describing virtual or augmented reality experiences aimed at clinicians working in the PICU.

*Blended learning*, which combines online learning and face-to-face learning, demonstrates superior knowledge acquisition compared to nonblended learning strategies (31). Two articles described blended learning strategies for teaching point-of-care ultrasound (POCUS). One article (9) provided a bundled educational intervention intended to be completed by pediatric residents during their PICU rotation with the support of supervising physicians competent in POCUS, including three modules for self-directed learning, pre- and post-curriculum test assessments, and guidelines for providing small group active learning experiences. The six residents who completed the curriculum reported satisfaction with the curriculum and demonstrated higher test scores compared to a peer cohort who completed the PICU rotation but not the POCUS curriculum, The second article (10) described the delivery of a multimodal curriculum to teach pediatric critical care transport nurses and paramedics how to perform POCUS targeted at confirming appropriate endotracheal tube position through both tracheal and thoracic ultrasound. The curriculum included videos, podcasts, readings, and videoconferencing sessions including case-based discussion and remote coaching and feedback on manual skills. Participants in this curriculum demonstrated high satisfaction with the curriculum, changes in attitudes with increased willingness to use POCUS, increased knowledge evidenced by increased test scores following the curriculum, and retention of test-based knowledge up to three months after the learning intervention. However, rates of post-intervention independent POCUS use during the study period were modest, increasing from 0 to 7.4% of all patients.

## 4. Discussion

In this study, we aimed to identify the current state of the evidence base for online education interventions targeted towards healthcare professionals working in the PICU. In our literature search, we identified eight manuscripts that describe online educational interventions targeted at the PICU context and patients. These manuscripts describe a variety of online learning modalities well represented in modern online education, including videos, websites, podcasts, videoconferencing, and screen-based simulation, as well as blended learning interventions leveraging multiple approaches to content delivery. The intended learners of the studies were residents (3 studies), nurses (2), multidisciplinary healthcare professionals (2), and transport nurses and paramedics (1). Most studies utilized participant surveys to assess satisfaction or change in attitudes, and half included pre- and post-intervention multiple-choice question tests. Only one study included a patient-related outcome measure.

We identified only eight manuscripts for inclusion through our screening process. Our search focused on a single pediatric subspecialty, but previously published evidence also suggests a low overall prevalence of reported studies of online educational interventions relating to pediatric subspecialties. Brusamento and colleagues (32) reported results of a systematic review and meta-analysis of the digital health professions education literature within all of pediatrics from 1990 to 2017 using Cochrane methodology. The authors screened 30,073 titles and abstracts and ultimately included fourteen studies in their final analysis that incorporated online learning. Thus, the number of published online learning manuscripts is low, even for the much larger specialty of pediatrics. We would likely expect lower rates for a subspecialty such as PICU.

Only one study included a patient-related outcome measure, and half of the studies included knowledge outcomes assessed by evaluating a change between pre- and post-test multiple question tests. These are outcome measures consistent with many educational studies in other disciplines and specialities (6). In fact, very few randomized controlled studies exist that assess the impact of online learning interventions compared to traditional teaching modalities for improving healthcare professionals' behaviors, knowledge or patient outcomes (33). Thus, health professionals educators continue to be challenged to conduct comparative effectiveness studies of differing educational interventions, and to assess clinician behaviors or patient outcomes (6, 34).

The growth of podcasts in medical education has been explosive (35, 36). It is notable that we only identified one study that included podcasts as part of the educational intervention in the pediatric critical care context. This likely reflects the relatively recent emergence of this technology in PICU education. Several PICU podcasts do exist, including PICU Doc on Call (37), PedsCrit (38), Paediatric Emergencies (39), Pediatrica Intensiva (40), PCICS (Pediatric Cardiac Intensive Care Society) Podcast (41), SCCM (Society of Critical Care Medicine) Podcast (42), and OPENPediatrics Podcasts (43). A recent review on podcasts in medical education by Kelly and colleagues (44) describes an increase in popularity of this modality in nearly all specialties, including highly visual specialties such as radiology. They report learner satisfaction with the modality, especially related to their flexibility, concise nature, and community building characteristics. As with many other online educational modalities, studies have demonstrated podcasts to be non-inferior when compared to more traditional modalities of teaching. More work is needed to best understand when and how to best use podcasts for medical education.

One limitation of our study includes our search strategy using MeSH terms. MeSH terms are manually assigned by the National Library of Medicine (https://www.nlm.nih.gov/mesh/meshhome.html), and given this process is not instantaneous, we may have missed some recently published articles. However, we estimate this limitation would only apply to a small number of the most-recently published articles. Additionally, MeSH terms may not exist for newly emerging topics. This seems less likely to be a significant limitation, as we did identify manuscripts that include relatively new learning modalities, including virtual and augmented reality-based interventions, in our initial search. None of those articles included education for healthcare professionals, and therefore were not included in this study. Another limitation includes our requirement that included manuscripts should describe educational interventions. Certainly many excellent online educational websites, videos, podcasts, courses, and other resources exist online, provide valuable contributions to education of PICU healthcare professionals, and have been described in the literature, but as our focus was on including studies of educational effectiveness, they fell outside of the scope of this study.

## 5. Conclusions

Although there is continued growth in the number and range of online educational efforts in many fields, including PICU, there are relatively few published studies in pediatric critical care, and only one study evaluated the impact of online learning on patient outcomes. Thus, there remain significant opportunities for PICU educators to design and conduct studies to assess the impact that online educational interventions can have in our field, especially related to clinician behaviors and patient outcomes.

## Data Availability

The raw data supporting the conclusions of this article will be made available by the authors, without undue reservation.
