# Supplementary material for: Online educational interventions in pediatric intensive care medicine
Source: Front Pediatr. 2023 Mar 9;11:1127754. doi: 10.3389/fped.2023.1127754 (PMC10033549; doi:10.3389/fped.2023.1127754)
Supplement: Supplementary file 1 [file Table1.docx]

Supplementary Table 1: The full list of MeSH terms used and number of search results returned.

| **Medical Subject Header Term** | **PICU paired search, number of results*** | **Critical care paired search, number of results**** |
| --- | --- | --- |
| Intensive Care Units, Pediatric | - | - |
| Education, Distance | 10 | 29 |
| Computer-Assisted Instruction | 12 | 79 |
| Computer Simulation | 50 | 225 |
| Simulation Training | 59 | 243 |
| Instructional film and video | 0 | 0 |
| Teaching | 120 | 602 |
| Gamification | 0 | 0 |
| Internet-Based Intervention | 3 | 0 |
| Curriculum | 38 | 742 |

*Each MeSH term was submitted to PubMed using the syntax (intensive care units, pediatric[MeSH Terms]) AND ([____[MeSH Terms])

**Each MeSH term was submitted to PubMed using the syntax (critical care[MeSH Terms]) AND ([____[MeSH Terms])
